# Neck Disability, Associated Factors, and Quality of Life Among Physiotherapists in Saudi Arabia

**DOI:** 10.3390/jcm15197390

**Published:** 2026-09-23

**Authors:** Ali S. Alqarni, Wael Alghamdi, Msaad Alzhrani

**Affiliations:** 1 Department of Physical Therapy and Health Rehabilitation, College of Applied Medical Sciences, Majmaah University, Al Majmaah 11952, Saudi Arabia; 441104324@s.mu.edu.sa; 2 Faculty of Nursing, Al-Baha University, Al-Baha 65779, Saudi Arabia; waelalghamdi@bu.edu.sa

**Keywords:** neck-related disability, Neck Disability Index, physiotherapists, quality of life, occupational health, Saudi Arabia

## Abstract

**Background/Objectives:** Physiotherapy practice involves patient handling, sustained or awkward postures, and repetitive manual techniques. This study estimated Neck Disability Index (NDI)-defined neck disability among physiotherapists in Saudi Arabia, examined its demographic and workplace associations, and compared quality of life across NDI levels. **Methods:** An online cross-sectional convenience survey (April–May 2024) included physiotherapists with ≥1 year of clinical experience. Disability and quality of life were assessed using the NDI and WHOQOL-BREF. Analyses used five-variable multinomial regression with robust standard errors, an expanded 11-factor mean bias-reduced sensitivity model, and Holm-adjusted comparisons. **Results:** Of 367 participants, 197 (53.7%; 95% CI, 48.6–58.7%) had NDI-defined disability: 156 (42.5%) mild and 41 (11.2%) moderate-or-worse. In the main model, mild disability was associated with female versus male sex (adjusted relative risk ratio [RRR], 2.49; 95% CI, 1.54–4.03) and body mass index (BMI) ≥25 versus <25 kg/m^2^ (RRR, 2.04; 95% CI, 1.26–3.29); moderate-or-worse disability was associated with pooled non-private versus private rehabilitation settings (RRR, 4.47; 95% CI, 2.06–9.73) and BMI ≥ 25 kg/m^2^ (RRR, 2.25; 95% CI, 1.05–4.84). The expanded model showed similar sex and workplace associations; only these factors were significant in Holm-adjusted joint tests, and the moderate-or-worse BMI estimate was less precise (RRR, 2.13; 95% CI, 0.96–4.69). All four WHOQOL-BREF domains showed lower medians at higher NDI levels (all omnibus Holm-adjusted *p* < 0.001), with the largest difference in physical health (ε^2^ = 0.199). **Conclusions:** Neck-related disability was common and co-occurred with poorer physical, psychological, social, and environmental quality of life. Sex and workplace showed the most consistent adjusted associations. These sample-specific findings warrant representative longitudinal research with direct measurement of occupational exposures and do not establish causality.

## 1. Introduction

Physiotherapists are exposed to a combination of patient handling, repetitive manual techniques, sustained trunk or neck positions, and prolonged standing. Systematic reviews have consistently identified work-related musculoskeletal disorders (WMSDs) as an important occupational-health concern in this profession [1,2,3,4]. A global meta-analysis of 26 studies estimated pooled neck-region musculoskeletal symptom prevalence at 26.4%, but heterogeneity was very high, reflecting differences in populations, recall periods, instruments, and prevalence denominators [2]. A more recent review likewise identified the lower back, neck, and thumb as frequently affected regions and emphasized the role of manual therapy, patient transfers, static postures, and workload [3]. An ergonomic observational study documented substantial biomechanical exposure during clinical physiotherapy, while a prospective cohort linked patient handling, bent or twisted postures, and manual techniques with subsequent WMSDs [5,6].

A plausible pathway from these exposures to neck-related disability involves repeated cervical flexion or rotation, sustained downward viewing of a treatment table, and forward trunk inclination during manual therapy or patient handling. These tasks may increase muscular demand and fatigue and accompany neck symptoms that interfere with activities such as lifting, reading, work, or sleep [5,6,7,8].

Neck pain contributes substantially to disability burden globally and in the Middle East and North Africa region [9,10]. Estimates among physiotherapists in Saudi Arabia and neighboring countries vary substantially. Saudi studies have reported neck-region WMSD estimates ranging from 26.6% to 59.2%, depending on the outcome definition and recall period [11,12]. Work-related musculoskeletal symptoms have also been reported among physiotherapists in Kuwait and the United Arab Emirates [13,14]. These studies establish that musculoskeletal complaints are common, but most measure symptom occurrence rather than the functional limitations attributable to neck symptoms.

Neck pain and neck-related disability are related but distinct constructs. Neck pain describes a symptom, whereas neck-related disability concerns perceived limitations in activities attributed to neck symptoms. The Neck Disability Index (NDI) combines symptom- and activity-related items, including personal care, lifting, reading, concentration, work, driving, sleep, and recreation [7,8]. Symptom prevalence alone cannot distinguish respondents with little interference in daily activities from those with more substantial limitations. NDI scores and severity categories therefore add a standardized description of functional burden to symptom reporting.

Combining a condition-specific patient-reported measure with a broader quality-of-life instrument can describe complementary aspects of health [15]. The WHOQOL-BREF was selected because it assesses physical health, psychological health, social relationships, and environment within one multidimensional instrument [16]. Comparing these domains across NDI levels allows neck-related functional burden to be considered alongside well-being, relationships, and the wider context of daily life. This provides a more comprehensive occupational-health description than physical symptoms alone, without assuming that neck-related disability causes differences in these domains.

Research among physiotherapists, other healthcare professionals, and general- or office-worker populations has examined demographic characteristics, workplace, physical workload, prolonged postures, computer use, psychosocial stress, and body composition in relation to neck symptoms or other WMSDs [1,5,12,17,18,19,20,21,22,23]. Nevertheless, evidence specifically describing NDI-defined neck-related disability and its relationship with quality of life among practicing physiotherapists in Saudi Arabia remains limited. This study therefore aimed to estimate the proportion of participating physiotherapists with NDI-defined neck-related disability, examine demographic and workplace associations with disability severity, and compare WHOQOL-BREF domain scores across NDI levels. We hypothesized that NDI severity would vary across selected demographic and workplace characteristics and would be associated with lower WHOQOL-BREF domain scores.

## 2. Materials and Methods

### 2.1. Study Design and Reporting

This cross-sectional study was reported with reference to the Strengthening the Reporting of Observational Studies in Epidemiology (STROBE) recommendations [24]. The survey was open from 2 April to 22 May 2024.

### 2.2. Recruitment and Participants

The survey link was distributed through email and professional networks using non-probability convenience sampling. The survey was administered using Google Forms (Google LLC, Mountain View, CA, USA), a web application. The invitation targeted physiotherapists practicing in Saudi Arabia, including those working in Ministry of Health hospitals and primary health centers, private medical rehabilitation centers, university hospitals, and military hospitals. Because distribution occurred through open electronic channels and the number of eligible physiotherapists who received or viewed the invitation was not tracked, a response rate could not be calculated.

Eligibility criteria were current physiotherapy practice in Saudi Arabia, at least one year of clinical experience, and electronic consent. Responses were excluded when required data were incomplete or the stated experience was less than one year. The invitation targeted licensed physiotherapists, but professional licensure was not independently verified by a questionnaire item.

### 2.3. Ethics

The Majmaah University Research Ethics Committee approved the study (registration H-01-R-088; ethics number MUREC-Mar.25/COM-2024/11-1; approval date 25 March 2024; expiry date 25 March 2025). Electronic informed consent was obtained before questionnaire completion.

### 2.4. Sample-Size Precision

A formal a priori sample-size calculation was not retained in the study records; accordingly, study adequacy is described using the achieved precision rather than a retrospectively reconstructed calculation. With 367 analyzed participants, the observed NDI ≥ 5 proportion of 53.7% had a two-sided 95% Wilson confidence interval of 48.6–58.7%, corresponding to an approximate half-width of 5.1 percentage points. This describes statistical precision only and does not correct for selection bias or establish national representativeness in a convenience sample.

### 2.5. Measures

The questionnaire collected age, sex, education, region, workplace, physiotherapy specialty, clinical experience, working hours per day, working days per week, BMI category, and smoking status. The collected variable was labeled “Sex” and offered male and female response categories; therefore, sex terminology is used consistently in this report.

The English NDI contains 10 sections scored from 0 to 5, yielding a total score from 0 to 50; higher scores indicate greater neck-related disability. The standard categories were no disability (0–4), mild disability (5–14), moderate disability (15–24), severe disability (25–34), and complete disability (35–50) [7,8]. These categories describe questionnaire-defined severity and are not clinical diagnoses or measures of fitness for work. Permission to use the NDI was obtained from the copyright holder through Mapi Research Trust.

The English WHOQOL-BREF contains 26 items. Following standard scoring, negatively framed items were reversed and domain means were multiplied by four to produce 4–20 scores for physical health, psychological health, social relationships, and environment; higher scores indicate better quality of life [16]. Permission to use the WHOQOL-BREF was obtained from the World Health Organization. The full copyrighted instruments are not reproduced in the manuscript or supplementary files.

### 2.6. Data Processing and Statistical Analysis

Of 411 study responses, one was excluded because required data were missing. The remaining 410-response spreadsheet was audited item by item. Forty-three responses from physiotherapists reporting less than one year of clinical experience were excluded, leaving 367 complete eligible responses. No score imputation was used. Category-label standardization is documented in Supplementary File S1. Exact duplicate response profiles were checked and none were identified; duplicate participation could not be definitively excluded because email capture was optional for most respondents. Internal consistency was summarized with Cronbach’s alpha.

The five standard NDI categories were retained for descriptive reporting. Wilson 95% confidence intervals were calculated for any disability (NDI ≥ 5) and for moderate-or-worse disability (NDI ≥ 15). Because severe and complete categories were sparse, association analyses used three levels: no disability, mild disability, and moderate-or-worse disability. Age and WHOQOL-BREF scores were compared with Kruskal–Wallis tests. Epsilon-squared was calculated as ε^2^ = max{0, (H − k + 1)/(n − k)}, where H is the Kruskal–Wallis statistic, k is the number of groups, and n is the total sample. Categorical variables were assessed with Pearson chi-square tests when expected counts were adequate; if any expected cell was <5, a Monte Carlo approximation to the Fisher–Freeman–Halton exact test with 200,000 samples was used. Cramér’s V was reported as the categorical effect size. Holm adjustment was applied across the 11 demographic/work comparisons and separately across the four WHOQOL-BREF domain tests.

Pairwise WHOQOL-BREF comparisons used two-sided Mann–Whitney tests with asymptotic, tie-adjusted *p* values and no continuity correction. Rank-biserial correlations summarize pairwise effect sizes; Holm adjustment was applied separately to the three comparisons within each domain (Supplementary Table S3).

A proportional-odds model was initially considered. A Brant-type global sandwich Wald test compared slopes from two cumulative binary logistic models, accounting for cross-equation dependence through observation-level influence functions. The slope-difference covariance matrix had rank 5, and the parallel-slopes restriction was not supported (χ^2^ = 15.02, df = 5, *p* = 0.010) [25]. Multinomial logistic regression therefore used no disability as the reference outcome and HC0 Huber–White robust standard errors. Following inspection of sparse cells, workplace was post hoc grouped as private medical rehabilitation versus non-private settings, and BMI as <25 versus ≥25 kg/m^2^, to reduce instability with only 41 moderate-or-worse observations. Sex, age, workplace, BMI group, and smoking were entered simultaneously.

The five-variable model provides an exploratory description of demographic characteristics (sex and age), employment context (workplace), and anthropometric and behavioral characteristics (BMI category and smoking). Its five slopes per outcome contrast limit complexity relative to the 32 slopes per contrast required by all 11 characteristics in their original categories. Covariate selection was not prospectively documented. Broader adjustment for education, region, specialty, clinical experience, hours/day, and days/week was assessed in the expanded sensitivity analysis; these characteristics are also reported in Supplementary Table S1 [24,26].

Adjusted relative risk ratios (RRRs) and 95% confidence intervals are reported. An RRR is the exponentiated multinomial coefficient and expresses the multiplicative change in the probability ratio of a specified disability category to no disability for a one-unit predictor change or category comparison, conditional on the other included variables. Individual coefficient *p* values and confidence intervals are nominal and exploratory. The expanded model separately reports joint factor tests with Holm adjustment across its 11 factors.

As the principal sensitivity analysis, a post hoc expanded multinomial model including all 11 measured demographic and work factors was fitted to the same 367 participants. The model included sex, continuous age, pooled workplace, pooled BMI, and smoking; clinical experience was grouped as ≥6 versus 1–5 years, working hours as >8 versus ≤8 h/day, and working days as 6–7 versus 1–5 days/week. These post hoc groupings limited additional parameters while preserving interpretable occupational contrasts. Education (five categories), specialty (eight categories), and region (five categories) used their original levels, with bachelor’s degree, orthopedic specialty, and Central Region as references. Age was centered at 28 years. The model estimated 23 slopes per outcome contrast (46 slopes and two intercepts), with all factors entered simultaneously and no stepwise or significance-based selection. Mean bias-reduced estimation used brglm2::brmultinom(type = “AS_mean”) to address sparse-data estimation and separation [27].

The expanded model used model-based standard errors from the Fisher information at the bias-reduced estimates, approximate Wald 95% confidence intervals, and two-sided nominal coefficient *p* values. Joint Wald tests evaluated each factor across both non-reference outcomes, with Holm adjustment across the 11 tests. Convergence, full design rank, finite estimates, and covariance calculations were checked (Supplementary Table S2).

Additional sensitivity analyses examined the original workplace and BMI categories, evaluated age using 2000 nonparametric bootstrap resamples and post hoc categories (21–26, 27–29, and 30–42 years), and included a centered quadratic age term. Exact bootstrap resampling indices are supplied. Statistical analyses were verified in R 4.6.1 (R Foundation for Statistical Computing, Vienna, Austria) using nnet 7.3-21, sandwich 3.1-3, MASS 7.3-66, and brglm2 1.1.0; complete software and execution records are provided in Supplementary File S4. The deidentified dataset and reproducible R code are also supplied. Two-sided *p* < 0.05 was considered statistically significant within the stated multiplicity framework.

### 2.7. Use of Generative Artificial Intelligence

ChatGPT (GPT-5.6 Sol initially, then GPT-6 Astra; OpenAI, San Francisco, CA, USA) assisted with language and readability editing, including rephrasing author-written text; document formatting; consistency checks; preparing and checking R analysis code; and preparing submission-support materials. The authors computed all statistical estimates using R code they reviewed, executed, and validated, and they were solely responsible for the study design, statistical methods, and interpretation of results. The authors reviewed and verified the final manuscript and take full responsibility for its content.

## 3. Results

### 3.1. Participant Flow and Characteristics

A total of 411 study responses were received. One response was excluded because required data were missing, and 43 responses were excluded because respondents reported less than one year of clinical experience. The analytic sample therefore comprised 367 participants (Figure 1).

Median age was 28 years (IQR, 26–30; range, 21–42), and 208 participants (56.7%) were female. Most held a bachelor’s degree (321, 87.5%), worked in private medical rehabilitation centers (230, 62.7%), practiced in orthopedics (180, 49.0%), and had one to five years of clinical experience (320, 87.2%) (Table 1).

The median NDI score was 5.0 (IQR, 2.0–10.0). Cronbach’s alpha was 0.844 for the NDI, 0.769 for WHOQOL-BREF physical health, 0.663 for psychological health, 0.787 for social relationships, and 0.852 for environment. The psychological-domain estimate was below 0.70 and is considered when interpreting that domain (Table 2).

### 3.2. NDI-Defined Neck-Related Disability

Overall, 197 participants had an NDI score ≥5 (53.7%; 95% CI, 48.6–58.7%). Mild disability was most common (156, 42.5%), followed by moderate disability (35, 9.5%), severe disability (5, 1.4%), and complete disability (1, 0.3%); 170 participants (46.3%) had no disability. In total, 41 participants had moderate-or-worse disability (NDI ≥15; 11.2%; 95% CI, 8.3–14.8%) (Figure 2).

### 3.3. Bivariate Associations

In the three-level analysis, sex was associated with NDI level (χ^2^ = 10.77, df = 2, raw *p* = 0.005, Cramér’s V = 0.171; Holm-adjusted *p* = 0.046). Among males, 44.0% had mild or moderate-or-worse disability, compared with 61.1% of females. Workplace was associated with NDI level (Fisher–Freeman–Halton Monte Carlo *p* < 0.001, Holm-adjusted *p* = 0.004, Cramér’s V = 0.211). BMI also showed an unadjusted association (Monte Carlo *p* = 0.006, Cramér’s V = 0.149), but its Holm-adjusted *p* value was 0.056. Age showed a small unadjusted difference across NDI levels (H = 6.01, raw *p* = 0.050, ε^2^ = 0.011) that did not remain significant after Holm adjustment (*p* = 0.396). Education, region, specialty, experience, working hours, working days, and smoking were not associated with NDI level after multiplicity adjustment (Table 3; Supplementary Table S1).

### 3.4. Adjusted Multinomial Analysis

The five-variable multinomial model converged and was significant overall (likelihood-ratio χ^2^ = 48.47, df = 10, *p* < 0.001; McFadden pseudo-R^2^ = 0.068). For mild disability versus no disability, positive adjusted associations were observed for female versus male sex (RRR, 2.49; 95% CI, 1.54–4.03; *p* < 0.001) and BMI ≥25 versus <25 kg/m^2^ (RRR, 2.04; 95% CI, 1.26–3.29; *p* = 0.004).

For moderate-or-worse disability versus no disability, positive adjusted associations were observed for non-private versus private rehabilitation employment (RRR, 4.47; 95% CI, 2.06–9.73; *p* < 0.001) and BMI ≥25 versus <25 kg/m^2^ (RRR, 2.25; 95% CI, 1.05–4.84; *p* = 0.037). Each additional year of age had an inverse association in the continuous-age model (RRR, 0.84; 95% CI, 0.74–0.95; *p* = 0.008), with a 2000-resample percentile bootstrap interval of 0.71–0.94. However, age was not significant after Holm adjustment in the bivariate analysis; the detailed original-category model yielded RRR 0.86 (95% CI, 0.74–1.00; *p* = 0.044), and categorical age estimates had confidence intervals crossing 1. Results are summarized in Table 4 and Supplementary Table S4.

The principal expanded 11-factor mean bias-reduced model converged with a full-rank design and finite estimates for all terms (n = 367; Supplementary Table S2). For mild versus no disability, the female sex estimate was RRR 2.66 (95% CI, 1.56–4.51; nominal *p* < 0.001), and the BMI ≥25 estimate was RRR 1.98 (95% CI, 1.17–3.34; nominal *p* = 0.011). For moderate-or-worse versus no disability, the non-private workplace estimate was RRR 5.58 (95% CI, 2.35–13.23; nominal *p* < 0.001). The BMI estimate remained similar to the five-variable result but was less precise (RRR, 2.13; 95% CI, 0.96–4.69; nominal *p* = 0.062); the age estimate was RRR 0.85 per year (95% CI, 0.74–0.99; nominal *p* = 0.031). Joint factor tests across both outcome contrasts remained significant after Holm adjustment for sex (*p* = 0.014) and workplace (*p* = 0.001), but not BMI (*p* = 0.216), age (*p* = 0.756), or the other factors. Several sparse education and specialty estimates were highly imprecise; lack of statistical evidence does not establish absence of an association. These findings support the consistency of the female sex/mild and workplace/moderate-or-worse patterns while requiring cautious interpretation of BMI, age, and sparse subgroup estimates.

The centered quadratic age term was not statistically significant in either outcome contrast (*p* = 0.265 for mild versus no disability and *p* = 0.311 for moderate-or-worse versus no disability), providing no clear evidence of age nonlinearity. The original-category sensitivity model produced point estimates that were not uniformly directional; their wide confidence intervals preclude firm conclusions about differences between individual categories. For mild disability, overweight BMI had an RRR of 2.52 (95% CI, 1.50–4.24), whereas obesity had an RRR of 0.35 (95% CI, 0.08–1.62) based on only 13 participants with obesity. Similarly, the mild-disability estimate for military hospitals was 0.55 (95% CI, 0.21–1.41), whereas the pooled non-private estimate was 1.10. Thus, the grouped BMI and workplace results are parsimonious model summaries that can mask non-monotonic or setting-specific patterns (Supplementary Table S2).

### 3.5. Quality of Life Across NDI Levels

WHOQOL-BREF domain medians decreased across higher NDI levels. The largest omnibus difference was observed for physical health (H = 74.29, Holm-adjusted *p* < 0.001, ε^2^ = 0.199), followed by psychological health (H = 41.15, adjusted *p* < 0.001, ε^2^ = 0.108), environment (H = 34.78, adjusted *p* < 0.001, ε^2^ = 0.090), and social relationships (H = 16.01, adjusted *p* < 0.001, ε^2^ = 0.038) (Table 5). Pairwise comparisons with within-domain Holm adjustment are provided in Supplementary Table S3. The mild versus moderate-or-worse contrast was significant for physical health (adjusted *p* = 0.002) and environment (adjusted *p* = 0.047), but not for psychological health (adjusted *p* = 0.136) or social relationships (adjusted *p* = 0.659).

## 4. Discussion

### 4.1. Principal Findings

In this convenience sample, 53.7% of physiotherapists had NDI-defined neck-related disability, predominantly mild, and 11.2% had moderate-or-worse disability. Female sex and BMI ≥ 25 kg/m^2^ were associated with mild disability in the five-variable model; non-private employment and BMI ≥ 25 kg/m^2^ were associated with moderate-or-worse disability. The expanded model showed similar sex and workplace associations, and only these factors had Holm-adjusted joint *p* < 0.05. Higher NDI levels co-occurred with lower scores across all four quality-of-life domains. BMI uncertainty, age sensitivity, and heterogeneity within pooled categories are considered below.

### 4.2. Comparison with Previous Research

The observed NDI-defined proportion measures neck-related functional burden and is not directly comparable with neck pain or WMSD symptom prevalence. The global physiotherapist meta-analysis estimated neck-region symptom prevalence at 26.4%, with substantial heterogeneity across instruments, recall periods, and denominators [2]. Saudi estimates of 26.6% and 59.2% and a UAE estimate of 28.7% similarly concern neck-region symptoms [11,12,14]. Previous NDI studies also describe substantial limitations among physiotherapists, although differences in samples and scoring limit direct numerical comparison [28,29].

The contribution of this study is the combined description of NDI-defined functional burden and multidimensional quality of life among physiotherapists in Saudi Arabia. Distinguishing predominantly mild disability from the smaller moderate-or-worse group conveys information that symptom prevalence alone would miss. The NDI captures perceived consequences for daily activities and work alongside symptoms [7,8]; item-level occupational performance outcomes were not analyzed. This extends the description provided by previous NDI research [28,29].

The female sex association with mild disability is broadly consistent with reports of greater musculoskeletal symptom burden among female physiotherapists [1,12], although the UAE study found no statistically significant sex difference [14]. In the present study, sex remained associated with NDI category after Holm correction in the bivariate analysis and in the expanded model’s joint test. The adjusted moderate-or-worse female sex estimate was imprecise, with a confidence interval including 1.

The association between pooled non-private employment and moderate-or-worse disability is compatible with prior observations concerning differences in workload, patient mix, equipment, direct-contact time, and organizational demands across settings [1,2,3,12]. The association was also observed in the expanded model. However, original-category estimates varied and were imprecise, so the pooled result summarizes heterogeneous settings (Supplementary Table S2). It supports directly measuring workplace exposures in future studies without establishing an occupational cause.

BMI ≥ 25 kg/m^2^ was associated with both severity contrasts in the five-variable model, whereas earlier Saudi WMSD research reported no BMI association [11]. Original-category estimates were non-monotonic and imprecise for obesity, precluding a dose–response interpretation. The expanded moderate-or-worse estimate was similar, but its confidence interval included 1 (RRR, 2.13; 95% CI, 0.96–4.69); the joint BMI test was not significant after Holm adjustment. The continuous-age association was model-dependent: findings varied across age specifications and multiplicity-adjusted tests despite bootstrap support. The young sample and small moderate-or-worse group limit firm age-related inference. Differences between the main and expanded models reflect changes in both adjustment and estimation method, so they cannot be attributed solely to additional covariates.

The predominance of respondents with 1–5 years of experience (320/367, 87.2%) is also relevant to occupational-health interpretation. Previous physiotherapist research has described WMSD onset early in professional practice, and qualitative work with final-year students highlights challenges in selecting feasible workplace-injury-prevention strategies [30,31]. Early-career clinicians may encounter frequent direct patient handling and substantial physical demands while developing practical ergonomic adaptations. Lower seniority may also limit control over caseload, breaks, task allocation, or choice of work setting. These are plausible contextual explanations, not measured characteristics of this cohort: ergonomic adaptation, clinical autonomy, and patient-handling frequency were not assessed. Moreover, clinical experience was not associated with NDI category in the bivariate analysis (Holm-adjusted *p* = 1.000), and relatively few participants had more than five years of experience. The sample composition therefore supports attention to early-career occupational health but does not demonstrate that less experience causes greater disability.

WHOQOL-BREF scores were lower at higher NDI levels, with the largest effect in physical health. This pattern is consistent with previous research reporting associations between WMSDs, lower quality of life, and workplace stress among physical therapists [32]. Some conceptual overlap is expected because the NDI assesses functional limitations while WHOQOL-BREF domains capture related dimensions of daily health and well-being. The psychological-domain finding should be interpreted with additional caution because its internal-consistency estimate was 0.663 in this sample. The findings demonstrate concurrent association, not that neck-related disability caused lower quality of life.

An earlier, non-peer-reviewed version of this study was posted on Preprints.org in March 2025 [33]. This report reanalyzes the same survey after data cleaning and verification of eligibility and questionnaire scoring. The preprint’s figure of 433 responses was a typographical error: 411 responses were received, and exclusion of one incomplete response and 43 respondents with less than the required year of clinical experience left 367 eligible participants. Statistical methods were revised to accommodate sparse data, model assumptions, and multiple comparisons, with broader adjustment and sensitivity analyses. The updated findings, including lower quality of life at higher NDI levels, supersede the preliminary report.

### 4.3. Strengths

Strengths include recruitment across all five Saudi regions and multiple workplace types, combined assessment of disability and multidimensional quality of life using established instruments, audited questionnaire scoring, and sensitivity analyses addressing broader adjustment and model stability. The supplied deidentified dataset, data dictionary, R code, exact bootstrap draws, and session information support reproducibility. Reporting follows STROBE recommendations [24].

### 4.4. Limitations and Implications

The study has several limitations. First, its cross-sectional design precludes causal or temporal inference. Second, open electronic convenience recruitment creates selection bias; the invitation denominator was unavailable, so response rate could not be calculated, and the sample cannot be assumed representative of all Saudi physiotherapists. Third, licensure was targeted in recruitment but not independently verified. Fourth, all measures were self-reported; BMI was recorded as a self-selected category rather than measured, no clinical examination was performed, and duplicate participation could not be completely excluded because most email fields were blank. The use of English-language instruments may also have favored respondents with greater English proficiency. Fifth, detailed ergonomic exposure, patient-handling frequency, caseload, psychosocial factors, physical activity, and relevant comorbidities were not measured. Sixth, the sample was young and concentrated in the one-to-five-year experience category, limiting comparisons across career stages. Some self-reported age–experience combinations were unusual; checking the original export confirmed the recorded values, but their accuracy could not be independently established. Seventh, sparse outcome and predictor categories required post hoc pooling, which can mask heterogeneity. Covariate selection was not prospectively documented, and broader adjustment in the expanded model does not eliminate residual confounding. The small moderate-or-worse group and sparse subgroups limited precision, with several wide intervals despite bias-reduced estimation; age estimates were sensitive to model specification. Eighth, internal consistency of the psychological WHOQOL-BREF domain was 0.663. Finally, overlap between neck-related disability content and WHOQOL-BREF domains may inflate some concurrent associations.

The results do not demonstrate the effectiveness of screening, ergonomic assessment, or occupational-health interventions. They identify questions for future evaluation. Probability-based or registry-supported sampling would improve representativeness; prospective cohorts should measure patient transfers, manual therapy exposure, posture, workload, psychosocial demands, and physical activity using standardized definitions. Intervention studies could then test whether organizational, ergonomic, educational, or workload modifications reduce incident neck symptoms and disability.

## 5. Conclusions

More than half of participating physiotherapists had NDI-defined neck-related disability, predominantly mild, and 11.2% had moderate-or-worse disability. Female sex and non-private workplace associations were consistent across the main and expanded models, while BMI and age findings require caution. Higher NDI levels co-occurred with lower quality of life across all four WHOQOL-BREF domains. These exploratory, sample-specific associations warrant representative longitudinal research with direct measurement of occupational exposures and do not establish causality.

## Figures and Tables

**Figure 1:**
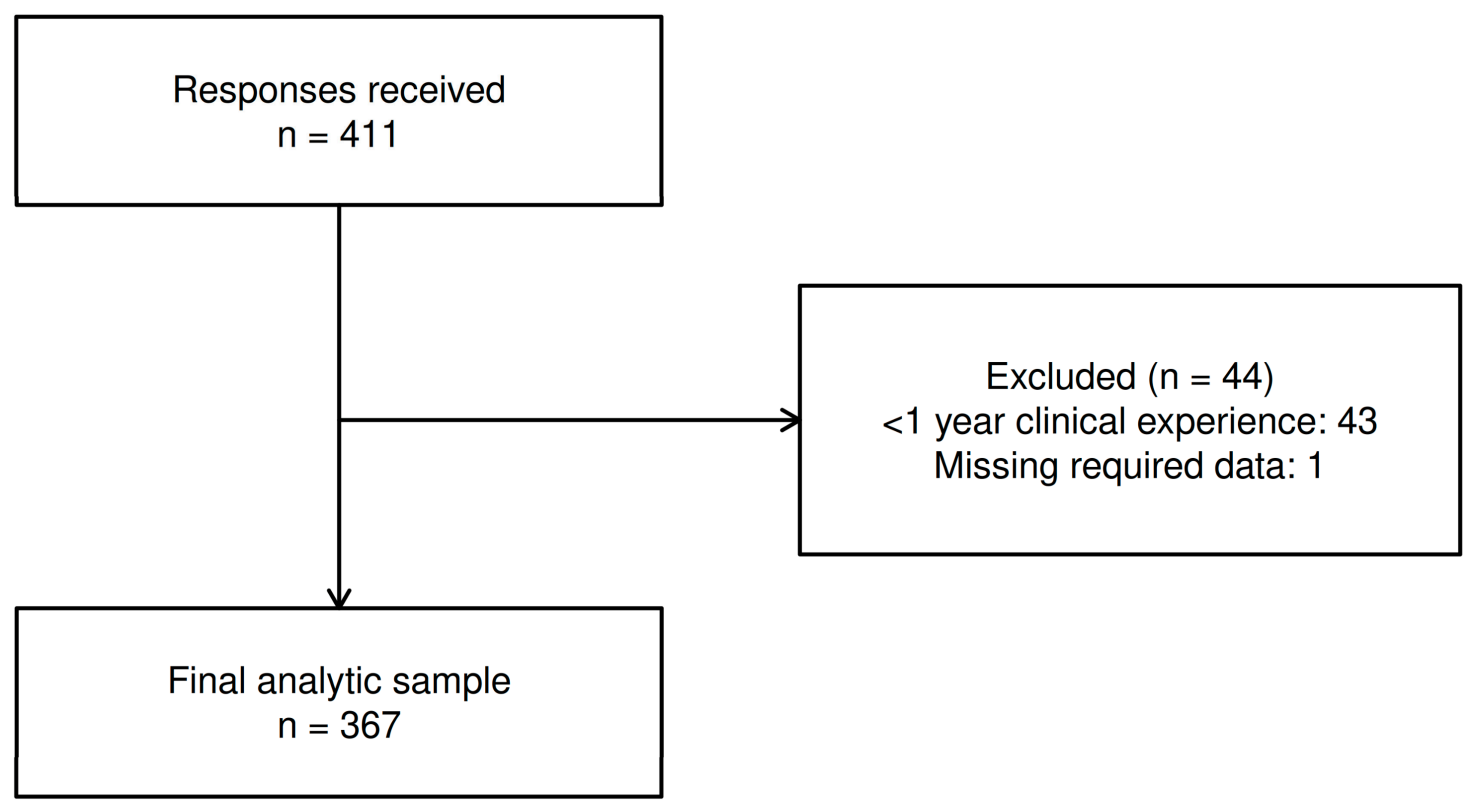
Participant flow. Of 411 responses received, 44 were excluded: one because required data were missing and 43 because respondents reported less than one year of clinical experience. The final analytic sample comprised 367 participants.

**Figure 2:**
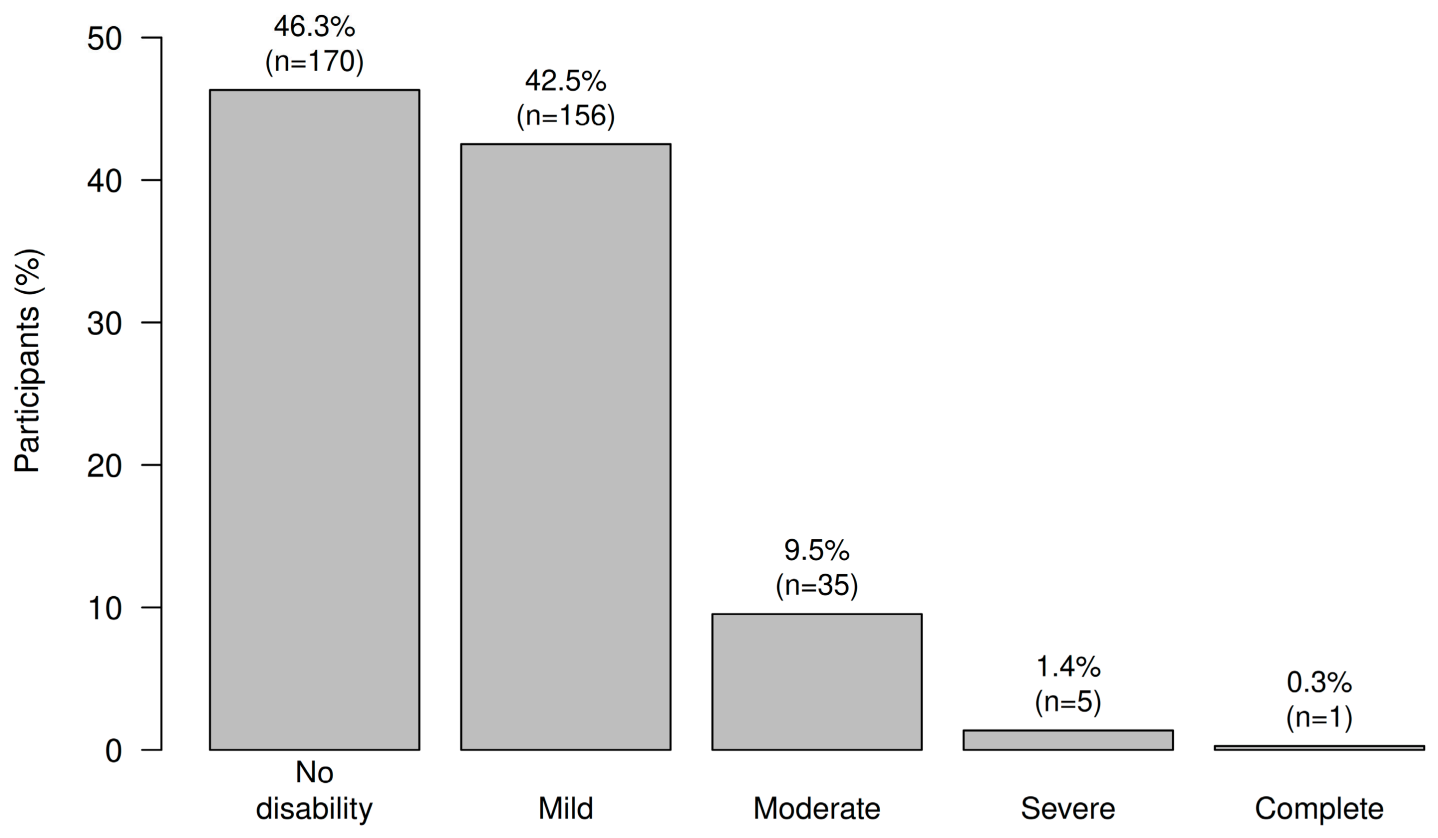
Distribution of the five standard Neck Disability Index (NDI) categories among 367 participating physiotherapists. Percentages are calculated using the final analytic sample as the denominator.

**Table 1:** Participant characteristics (n = 367).

Characteristic	Category	n (%) or Summary
Age, years	Median (IQR); range	28 (26–30); 21–42
Sex	Male	159 (43.3%)
	Female	208 (56.7%)
Education	Diploma	4 (1.1%)
	Bachelor’s degree	321 (87.5%)
	Completed some postgraduate	8 (2.2%)
	Master’s degree	32 (8.7%)
	PhD	2 (0.5%)
Region	Central Region	98 (26.7%)
	Eastern Region	40 (10.9%)
	Western Region	100 (27.2%)
	Southern Region	88 (24.0%)
	Northern Region	41 (11.2%)
Workplace	Private medical rehabilitation centers	230 (62.7%)
	Ministry of Health hospitals	80 (21.8%)
	Primary health centers (Ministry of Health)	15 (4.1%)
	University hospitals	16 (4.4%)
	Military hospitals	26 (7.1%)
PT specialty	Orthopedic	180 (49.0%)
	Neurology	55 (15.0%)
	Pediatric	66 (18.0%)
	Geriatrics	14 (3.8%)
	Cardiothoracic	6 (1.6%)
	Oncology	7 (1.9%)
	General physical therapy	35 (9.5%)
	Musculoskeletal	4 (1.1%)
Clinical experience	1–5 years	320 (87.2%)
	6–9 years	33 (9.0%)
	≥10 years	14 (3.8%)
Working hours/day	<5 h	21 (5.7%)
	5–8 h	291 (79.3%)
	9–12 h	50 (13.6%)
	>12 h	5 (1.4%)
Working days/week	1–3 days	17 (4.6%)
	4–5 days	192 (52.3%)
	6–7 days	158 (43.1%)
BMI category	Underweight	24 (6.5%)
	Normal weight	224 (61.0%)
	Overweight	106 (28.9%)
	Obese	13 (3.5%)
Smoking	No	311 (84.7%)
	Yes	56 (15.3%)

BMI, body mass index. Age is presented as median (IQR) and range; categorical variables as n (%).

**Table 2:** NDI and WHOQOL-BREF score distributions.

Measure	Median (IQR)	Range	Cronbach α
NDI total score	5.0 (2.0–10.0)	0.0–35.0	0.844
WHOQOL-BREF physical	14.3 (12.0–16.6)	7.4–20.0	0.769
WHOQOL-BREF psychological	13.3 (11.3–15.3)	6.7–20.0	0.663
WHOQOL-BREF social relationships	13.3 (12.0–16.0)	4.0–20.0	0.787
WHOQOL-BREF environment	13.0 (11.0–15.5)	4.0–20.0	0.852

NDI, Neck Disability Index. WHOQOL-BREF domain scores use the 4–20 scale; higher scores indicate better quality of life. The psychological-domain alpha (0.663) is below the conventional 0.70 benchmark and is interpreted cautiously.

**Table 3:** Bivariate associations with three-level NDI severity.

Variable	Test	Statistic	df	Raw *p*	Holm *p*	Effect Size	Min Expected
Age	Kruskal–Wallis	H = 6.01	2	0.050	0.396	ε^2^ = 0.011	
Sex	Pearson chi-square	χ^2^ = 10.77	2	0.005	0.046	Cramér V = 0.171	17.76
Education	Fisher–Freeman–Halton Monte Carlo	χ^2^ = 8.68	8	0.359	1.000	Cramér V = 0.109	0.22
Region	Fisher–Freeman–Halton Monte Carlo	χ^2^ = 9.60	8	0.326	1.000	Cramér V = 0.114	4.47
Workplace	Fisher–Freeman–Halton Monte Carlo	χ^2^ = 32.54	8	<0.001	0.004	Cramér V = 0.211	1.68
PT specialty	Fisher–Freeman–Halton Monte Carlo	χ^2^ = 14.55	14	0.383	1.000	Cramér V = 0.141	0.45
Clinical experience	Fisher–Freeman–Halton Monte Carlo	χ^2^ = 0.37	4	0.974	1.000	Cramér V = 0.023	1.56
Working hours/day	Fisher–Freeman–Halton Monte Carlo	χ^2^ = 2.05	6	0.874	1.000	Cramér V = 0.053	0.56
Working days/week	Fisher–Freeman–Halton Monte Carlo	χ^2^ = 1.64	4	0.759	1.000	Cramér V = 0.047	1.90
BMI category	Fisher–Freeman–Halton Monte Carlo	χ^2^ = 16.28	6	0.006	0.056	Cramér V = 0.149	1.45
Smoking	Pearson chi-square	χ^2^ = 1.82	2	0.403	1.000	Cramér V = 0.070	6.26

NDI outcome: no disability, mild disability, and moderate-or-worse disability. Pearson χ^2^ was used only when expected counts were adequate; otherwise, a Fisher–Freeman–Halton test with 200,000 Monte Carlo samples was used. Holm adjustment covers the 11 demographic/work comparisons. ε^2^ = max{0, (H – k + 1)/(n − k)}.

**Table 4:** Adjusted multinomial logistic regression for NDI severity.

Outcome Contrast	Predictor	Adjusted RRR (95% CI)	z	*p*
Mild vs. no disability	Female vs. male	2.49 (1.54–4.03)	3.71	<0.001
Mild vs. no disability	Age, per year	0.99 (0.92–1.07)	−0.17	0.866
Mild vs. no disability	Non-private vs. private rehabilitation setting	1.10 (0.68–1.77)	0.40	0.692
Mild vs. no disability	BMI ≥25 vs. <25 kg/m^2^	2.04 (1.26–3.29)	2.90	0.004
Mild vs. no disability	Smoking: yes vs. no	1.59 (0.81–3.10)	1.35	0.176
Moderate-or-worse vs. no disability	Female vs. male	1.83 (0.80–4.21)	1.43	0.153
Moderate-or-worse vs. no disability	Age, per year	0.84 (0.74–0.95)	−2.66	0.008
Moderate-or-worse vs. no disability	Non-private vs. private rehabilitation setting	4.47 (2.06–9.73)	3.78	<0.001
Moderate-or-worse vs. no disability	BMI ≥25 vs. <25 kg/m^2^	2.25 (1.05–4.84)	2.08	0.037
Moderate-or-worse vs. no disability	Smoking: yes vs. no	2.43 (0.86–6.85)	1.67	0.094

Reference outcome: no disability. RRR, relative risk ratio. HC0 Huber–White model-robust sandwich standard errors. Predictor pooling of workplace and BMI was post hoc because of sparse cells and the limited moderate-or-worse event count. All listed predictors were entered simultaneously. Adjustment is limited to these five variables; individual *p* values and 95% confidence intervals are nominal, without multiplicity adjustment. The principal expanded 11-factor bias-reduced sensitivity analysis is reported in Supplementary Table S2.

**Table 5:** WHOQOL-BREF domains across NDI severity levels.

Domain	No Disability Median (IQR)	Mild Median (IQR)	Moderate-or-Worse Median (IQR)	H	Holm *p*	ε^2^
Physical health	16.0 (13.7–17.1)	13.1 (12.0–14.9)	12.0 (10.9–13.1)	74.29	<0.001	0.199
Psychological health	14.7 (12.7–16.0)	12.7 (10.7–14.7)	12.0 (10.7–13.3)	41.15	<0.001	0.108
Social relationships	14.7 (12.0–16.0)	13.3 (10.7–16.0)	12.0 (10.7–14.7)	16.01	<0.001	0.038
Environment	14.5 (12.5–16.0)	12.2 (10.5–15.0)	11.5 (10.5–13.0)	34.78	<0.001	0.090

WHOQOL-BREF domain scores are on the 4–20 scale; higher scores indicate better quality of life. Holm adjustment covers the four domain-level Kruskal–Wallis tests. ε^2^ = max{0, (H − k + 1)/(n − k)}.

## Data Availability

The deidentified numeric analysis dataset (Data S1), data dictionary (File S1), reproducible R analysis script (Code S1), archived bootstrap coefficients (File S2), exact 2000-resample bootstrap indices (File S3), expanded-model coefficient and joint-test outputs (Files S5 and S6), and R session information (File S4) are provided with this submission. Copyrighted questionnaire wording, timestamps, email addresses, and free-text fields are not included. The original survey export is not publicly shared because it contains potentially identifying information and copyrighted instrument text.

## Supplementary Materials

The following supporting information can be downloaded at: https://www.mdpi.com/article/10.3390/jcm15197390/s1, Table S1: Full demographic and workplace cross-tabulations by NDI severity; Table S2: Sensitivity analyses of the adjusted multinomial model [27]; Table S3: Pairwise WHOQOL-BREF comparisons between NDI levels; Table S4: Age-stability analyses; Data S1: Deidentified analysis dataset; File S1: Data dictionary; Code S1: Reproducible R analysis; File S2: Age bootstrap log coefficients; File S3: Age bootstrap indices (2000 × 367); File S4: R session information; File S5: Expanded-model coefficients; File S6: Expanded-model joint factor tests.
