# Supplementary material for: Neck Disability, Associated Factors, and Quality of Life Among Physiotherapists in Saudi Arabia
Source: J Clin Med. 2026 Sep 23;15(19):7390. doi: 10.3390/jcm15197390 (PMC13665763; doi:10.3390/jcm15197390)
Supplement: Supplementary file 1 [file jcm-15-07390-s001.zip › File_S1_Data_Dictionary.pdf]

## Supplementary File S1. Data dictionary

Copyrighted NDI and WHOQOL-BREF item wording is intentionally not reproduced.

Before the Data S1 export was prepared, trailing spaces in source-data category labels were standardized, and the single “General” specialty entry was harmonized with “General physical therapy.” These historical cleaning steps did not change the analysis sample: one response with missing required data and 43 responses from physiotherapists with less than one year of clinical experience were excluded from the 411 responses received, leaving 367 complete eligible participants. No missing scores were imputed. Data S1 contains the resulting coded analysis data.

| Variable | Description | Coding / range |
|---|---|---|
| study_id | Synthetic sequential study identifier; not an original participant identifier. | 1–367 |
| sex_code | Sex as collected in the survey. | 0=Male; 1=Female |
| age_years | Age in years. | Numeric |
| education_code | Highest education category. | 1=Diploma; 2=Bachelor’s degree; 3=Some postgraduate; 4=Master’s degree; 5=PhD |
| region_code | Saudi region. | 1=Central; 2=Eastern; 3=Western; 4=Southern; 5=Northern |
| workplace_code | Workplace category. | 1=Private medical rehabilitation center; 2=Ministry of Health hospital; 3=Ministry of Health primary health center; 4=University hospital; 5=Military hospital |
| specialty_code | Physiotherapy practice specialty. | 1=Orthopedic; 2=Neurology; 3=Pediatric; 4=Geriatrics; 5=Cardiothoracic; 6=Oncology; 7=General physical therapy; 8=Musculoskeletal |
| experience_code | Clinical experience category. | 1=1–5 years; 2=6–9 years; 3=≥10 years |
| hours_code | Working hours per day. | 1=<5; 2=5–8; 3=9–12; 4=>12 |
| days_code | Working days per week. | 1=1–3; 2=4–5; 3=6–7 |
| bmi_code | Self-reported BMI category. | 1=Underweight; 2=Normal weight; 3=Overweight; 4=Obese |
| smoking_code | Smoking status. | 0=No; 1=Yes |
| NDI_01 | Numeric response to NDI item; copyrighted item wording is not reproduced. | 0–5 |
| NDI_02 | Numeric response to NDI item; copyrighted item wording is not reproduced. | 0–5 |
| NDI_03 | Numeric response to NDI item; copyrighted item wording is not reproduced. | 0–5 |
| NDI_04 | Numeric response to NDI item; copyrighted item wording is not reproduced. | 0–5 |
| NDI_05 | Numeric response to NDI item; copyrighted item wording is not reproduced. | 0–5 |
| NDI_06 | Numeric response to NDI item; copyrighted item wording is not reproduced. | 0–5 |
| NDI_07 | Numeric response to NDI item; copyrighted item wording is not reproduced. | 0–5 |
| NDI_08 | Numeric response to NDI item; copyrighted item wording is not reproduced. | 0–5 |
| NDI_09 | Numeric response to NDI item; copyrighted item wording is not reproduced. | 0–5 |
| NDI_10 | Numeric response to NDI item; copyrighted item wording is not reproduced. | 0–5 |
| WHO_01 | Numeric response to WHOQOL-BREF item; copyrighted item wording is not reproduced. | 1–5 |
| WHO_02 | Numeric response to WHOQOL-BREF item; copyrighted item wording is not reproduced. | 1–5 |
| WHO_03 | Numeric response to WHOQOL-BREF item; copyrighted item wording is not reproduced. | 1–5 |
| WHO_04 | Numeric response to WHOQOL-BREF item; copyrighted item wording is not reproduced. | 1–5 |
| WHO_05 | Numeric response to WHOQOL-BREF item; copyrighted item wording is not reproduced. | 1–5 |
| WHO_06 | Numeric response to WHOQOL-BREF item; copyrighted item wording is not reproduced. | 1–5 |
| WHO_07 | Numeric response to WHOQOL-BREF item; copyrighted item wording is not reproduced. | 1–5 |
| WHO_08 | Numeric response to WHOQOL-BREF item; copyrighted item wording is not reproduced. | 1–5 |
| WHO_09 | Numeric response to WHOQOL-BREF item; copyrighted item wording is not reproduced. | 1–5 |
| WHO_10 | Numeric response to WHOQOL-BREF item; copyrighted item wording is not reproduced. | 1–5 |
| WHO_11 | Numeric response to WHOQOL-BREF item; copyrighted item wording is not reproduced. | 1–5 |
| WHO_12 | Numeric response to WHOQOL-BREF item; copyrighted item wording is not reproduced. | 1–5 |
| WHO_13 | Numeric response to WHOQOL-BREF item; copyrighted item wording is not reproduced. | 1–5 |
| WHO_14 | Numeric response to WHOQOL-BREF item; copyrighted item wording is not reproduced. | 1–5 |
| WHO_15 | Numeric response to WHOQOL-BREF item; copyrighted item wording is not reproduced. | 1–5 |

| Variable | Description | Coding / range |
|---|---|---|
| WHO_16 | Numeric response to WHOQOL-BREF item; copyrighted item wording is not reproduced. | 1–5 |
| WHO_17 | Numeric response to WHOQOL-BREF item; copyrighted item wording is not reproduced. | 1–5 |
| WHO_18 | Numeric response to WHOQOL-BREF item; copyrighted item wording is not reproduced. | 1–5 |
| WHO_19 | Numeric response to WHOQOL-BREF item; copyrighted item wording is not reproduced. | 1–5 |
| WHO_20 | Numeric response to WHOQOL-BREF item; copyrighted item wording is not reproduced. | 1–5 |
| WHO_21 | Numeric response to WHOQOL-BREF item; copyrighted item wording is not reproduced. | 1–5 |
| WHO_22 | Numeric response to WHOQOL-BREF item; copyrighted item wording is not reproduced. | 1–5 |
| WHO_23 | Numeric response to WHOQOL-BREF item; copyrighted item wording is not reproduced. | 1–5 |
| WHO_24 | Numeric response to WHOQOL-BREF item; copyrighted item wording is not reproduced. | 1–5 |
| WHO_25 | Numeric response to WHOQOL-BREF item; copyrighted item wording is not reproduced. | 1–5 |
| WHO_26 | Numeric response to WHOQOL-BREF item; copyrighted item wording is not reproduced. | 1–5 |
| ndi_total | Recomputed NDI total score. | 0–50 |
| ndi5_category_code | Five-category NDI classification. | 0=No; 1=Mild; 2=Moderate; 3=Severe; 4=Complete disability |
| ndi3_category_code | Three-level analysis outcome. | 0=No; 1=Mild; 2=Moderate-or-worse disability |
| who_physical_4_20 | WHOQOL-BREF physical-health domain score | 4–20; higher=better quality of life |
| who_psychological_4_20 | WHOQOL-BREF psychological-health domain score | 4–20; higher=better quality of life |
| who_social_4_20 | WHOQOL-BREF social-relationships domain score | 4–20; higher=better quality of life |
| who_environment_4_20 | WHOQOL-BREF environment domain score | 4–20; higher=better quality of life |
