# Supplementary material for: Neck Disability, Associated Factors, and Quality of Life Among Physiotherapists in Saudi Arabia
Source: J Clin Med. 2026 Sep 23;15(19):7390. doi: 10.3390/jcm15197390 (PMC13665763; doi:10.3390/jcm15197390)
Supplement: Supplementary file 1 [file jcm-15-07390-s001.zip › Table_S1_Full_Crosstabs.pdf]

Supplementary Table S1. Full demographic and workplace cross-tabulations by NDI severity

Percentages are within each predictor category. This table presents original categories; sparse cells are apparent and motivated exact testing and post hoc pooling in the adjusted parsimonious model.

Variable	Category	Total n	No disability n (%)	Mild n (%)	Moderate-or-worse n (%)
Sex	Male	159	89 (56.0%)	54 (34.0%)	16 (10.1%)
	Female	208	81 (38.9%)	102 (49.0%)	25 (12.0%)
Education	Diploma	4	0 (0.0%)	3 (75.0%)	1 (25.0%)
	Bachelor's degree	321	153 (47.7%)	131 (40.8%)	37 (11.5%)
	Completed some postgraduate	8	3 (37.5%)	5 (62.5%)	0 (0.0%)
	Master's degree	32	14 (43.8%)	15 (46.9%)	3 (9.4%)
	PhD	2	0 (0.0%)	2 (100.0%)	0 (0.0%)
Region	Central Region	98	39 (39.8%)	49 (50.0%)	10 (10.2%)
	Eastern Region	40	23 (57.5%)	14 (35.0%)	3 (7.5%)
	Western Region	100	46 (46.0%)	43 (43.0%)	11 (11.0%)
	Southern Region	88	47 (53.4%)	32 (36.4%)	9 (10.2%)
	Northern Region	41	15 (36.6%)	18 (43.9%)	8 (19.5%)
Workplace	Private medical rehabilitation centers	230	114 (49.6%)	102 (44.3%)	14 (6.1%)
	Ministry of Health hospitals	80	33 (41.2%)	36 (45.0%)	11 (13.8%)
	Primary health centers (Ministry of Health)	15	4 (26.7%)	5 (33.3%)	6 (40.0%)
	University hospitals	16	5 (31.2%)	5 (31.2%)	6 (37.5%)
	Military hospitals	26	14 (53.8%)	8 (30.8%)	4 (15.4%)
PT specialty	Orthopedic	180	89 (49.4%)	73 (40.6%)	18 (10.0%)
	Neurology	55	20 (36.4%)	28 (50.9%)	7 (12.7%)
	Pediatric	66	29 (43.9%)	29 (43.9%)	8 (12.1%)
	Geriatrics	14	6 (42.9%)	4 (28.6%)	4 (28.6%)
	Cardiothoracic	6	1 (16.7%)	4 (66.7%)	1 (16.7%)
	Oncology	7	3 (42.9%)	3 (42.9%)	1 (14.3%)
	General physical therapy	35	21 (60.0%)	12 (34.3%)	2 (5.7%)
	Musculoskeletal	4	1 (25.0%)	3 (75.0%)	0 (0.0%)
Clinical experience	1–5 years	320	149 (46.6%)	135 (42.2%)	36 (11.2%)
	6–9 years	33	15 (45.5%)	15 (45.5%)	3 (9.1%)
	≥10 years	14	6 (42.9%)	6 (42.9%)	2 (14.3%)
Working hours/day	<5 hours	21	10 (47.6%)	9 (42.9%)	2 (9.5%)
	5–8 hours	291	133 (45.7%)	123 (42.3%)	35 (12.0%)
	9–12 hours	50	25 (50.0%)	22 (44.0%)	3 (6.0%)
	>12 hours	5	2 (40.0%)	2 (40.0%)	1 (20.0%)
Working days/week	1–3 days	17	9 (52.9%)	5 (29.4%)	3 (17.6%)
	4–5 days	192	87 (45.3%)	84 (43.8%)	21 (10.9%)
	6–7 days	158	74 (46.8%)	67 (42.4%)	17 (10.8%)
BMI category	Underweight	24	11 (45.8%)	9 (37.5%)	4 (16.7%)
	Normal weight	224	116 (51.8%)	87 (38.8%)	21 (9.4%)
	Overweight	106	34 (32.1%)	58 (54.7%)	14 (13.2%)
	Obese	13	9 (69.2%)	2 (15.4%)	2 (15.4%)
Smoking	No	311	147 (47.3%)	132 (42.4%)	32 (10.3%)
	Yes	56	23 (41.1%)	24 (42.9%)	9 (16.1%)
