# Supplementary material for: Neck Disability, Associated Factors, and Quality of Life Among Physiotherapists in Saudi Arabia
Source: J Clin Med. 2026 Sep 23;15(19):7390. doi: 10.3390/jcm15197390 (PMC13665763; doi:10.3390/jcm15197390)
Supplement: Supplementary file 1 [file jcm-15-07390-s001.zip › Table_S2_Detailed_Sensitivity_Model.pdf]

Supplementary Table S2. Sensitivity analyses of the adjusted multinomial model

All analyses use the same 367 eligible complete responses: 170 with no disability, 156 with mild disability, and 41 with moderate-or-worse disability. Part A presents the principal post hoc expanded sensitivity analysis; Part D presents the sensitivity results using original workplace and BMI categories. These are exploratory conditional associations, not prospective risk estimates or causal effects.

Model specification and inference

The expanded model includes sex, continuous age centered at 28 years, pooled workplace (non-private versus private rehabilitation), pooled BMI (≥25 versus <25 kg/m²), and smoking. It additionally includes clinical experience (≥6 versus 1–5 years), hours/day (>8 versus ≤8), days/week (6–7 versus 1–5), education (five original levels), specialty (eight original levels), and region (five original levels). Education, specialty, and region reference levels are bachelor's degree, orthopedic specialty, and Central region. Occupational groupings were made post hoc for interpretable contrasts with fewer parameters; no stepwise or significance-based screening selected factors for this expanded model. Age is reported per one-year increase. All 11 factors are entered simultaneously: 23 slopes per contrast, 46 slopes and two intercepts in total.

Estimation uses mean bias-reduced multinomial logistic regression, brglm2::brmultinom(type = "AS_mean"), with no disability as the reference outcome. Individual coefficient intervals and *p* values are nominal approximate Wald quantities using the model-based Fisher information at the bias-reduced estimates; they are not HC0 or profile-likelihood intervals. Part B provides joint Wald tests across both outcome contrasts, with Holm adjustment across the 11 factors. This correction does not apply to the coefficient intervals and is not a study-wide correction across every analysis. The package and software sessions are recorded in File S4; Code S1 reproduces the analysis.

The model converged in 11 iterations with a full-rank design (rank 24), finite coefficients, and a maximum absolute penalized-score numerical gradient of 9.70×10^{-9}. Independently reconstructed Fisher information and the package covariance agreed to within 8.0×10^{-15}. These checks support numerical computation, but do not establish sufficient information for every subgroup: education includes diploma n = 4 and PhD n = 2, and several specialties have fewer than ten participants. Very wide intervals must be interpreted accordingly.

Part A. Principal expanded 11-factor bias-reduced estimates

Predictor comparison	Mild versus no disability: RRR (95% CI)	Nominal *p*	Moderate-or-worse versus no disability: RRR (95% CI)	Nominal *p*
Female versus male	2.66 (1.56–4.51)	<0.001	1.41 (0.63–3.13)	0.402
Age, per year	0.98 (0.89–1.07)	0.601	0.85 (0.74–0.99)	0.031
Non-private versus private rehabilitation	0.93 (0.52–1.66)	0.795	5.58 (2.35–13.23)	<0.001
BMI ≥25 versus <25 kg/m²	1.98 (1.17–3.34)	0.011	2.13 (0.96–4.69)	0.062
Smoker versus non-smoker	1.68 (0.82–3.48)	0.159	2.37 (0.88–6.37)	0.086
Experience ≥6 versus 1–5 years	1.15 (0.48–2.75)	0.749	1.25 (0.33–4.65)	0.741

**Predictor comparison**	**Mild versus no disability: RRR (95% CI)**	**Nominal *p***	**Moderate-or-worse versus no disability: RRR (95% CI)**	**Nominal *p***
Hours/day >8 versus ≤8	0.86 (0.44–1.69)	0.667	0.49 (0.16–1.55)	0.227
Days/week 6–7 versus 1–5	1.04 (0.61–1.80)	0.876	2.13 (0.91–4.94)	0.080
Diploma versus bachelor's degree	11.09 (0.25–488.55)	0.213	4.39 (0.05–356.81)	0.509
Some postgraduate study versus bachelor's degree	1.55 (0.31–7.72)	0.591	0.40 (0.01–12.57)	0.603
Master's versus bachelor's degree	1.16 (0.48–2.79)	0.741	1.00 (0.26–3.92)	0.994
PhD versus bachelor's degree	6.56 (0.09–463.44)	0.386	2.29 (0.01–500.50)	0.763
Neurology versus orthopedic	1.63 (0.81–3.30)	0.174	1.64 (0.57–4.68)	0.356
Pediatric versus orthopedic	1.18 (0.60–2.32)	0.634	2.12 (0.76–5.93)	0.151
Geriatrics versus orthopedic	0.65 (0.16–2.62)	0.545	2.06 (0.45–9.51)	0.354
Cardiothoracic versus orthopedic	5.43 (0.54–54.31)	0.150	4.53 (0.26–78.69)	0.300
Oncology versus orthopedic	1.16 (0.20–6.69)	0.870	1.11 (0.10–12.41)	0.933
General physical therapy versus orthopedic	0.71 (0.31–1.62)	0.415	0.50 (0.11–2.17)	0.355
Musculoskeletal versus orthopedic	4.46 (0.41–48.69)	0.220	1.80 (0.03–95.98)	0.773
Eastern versus Central region	0.70 (0.29–1.65)	0.411	0.99 (0.24–4.11)	0.991
Western versus Central region	0.91 (0.47–1.77)	0.787	1.33 (0.47–3.72)	0.588
Southern versus Central region	0.69 (0.35–1.36)	0.285	0.70 (0.24–2.07)	0.523
Northern versus Central region	1.09 (0.45–2.64)	0.845	1.89 (0.58–6.19)	0.294

RRR, relative risk ratio; CI, confidence interval. The full coefficient CSV (File S5) includes both intercepts, log coefficients, standard errors, RRRs, intervals, and nominal *p* values. No individual interval is adjusted for multiplicity.

Part B. Joint factor tests across both outcome contrasts

**Factor**	**Degrees of freedom**	**Wald chi-square**	**Raw *p***	**Holm-adjusted *p***
Sex	2	13.138	0.001	0.014
Age	2	4.719	0.094	0.756
Workplace	2	17.956	<0.001	0.001
BMI group	2	7.457	0.024	0.216
Smoking	2	3.599	0.165	1.000
Clinical experience	2	0.155	0.925	1.000
Hours/day	2	1.465	0.481	1.000
Days/week	2	3.248	0.197	1.000
Education	8	3.364	0.909	1.000
Specialty	14	11.156	0.674	1.000
Region	8	4.289	0.830	1.000

The machine-readable joint-test results are provided in File S6. A joint test evaluates the factor across mild and moderate-or-worse contrasts together. A significant joint test does not establish significance for every individual category or outcome contrast. Sex and workplace have significant joint tests after this 11-test Holm correction; the remaining factors do not. Nonsignificance does not demonstrate absence of an association, particularly for sparse categories.

Part C. Interpretation relative to the five-variable main model

The female-sex estimate for mild disability is 2.66 (95% CI, 1.56–4.51), compared with 2.49 (1.54–4.03) in the main model. The pooled workplace estimate for moderate-or-worse disability is 5.58 (2.35–13.23), compared with 4.47 (2.06–9.73). The moderate-or-worse BMI estimate is 2.13 (0.96–4.69), compared with 2.25 (1.05–4.84): the point estimate is similar, but its confidence interval crosses 1. The mild BMI contrast remains nominally significant, but the joint BMI test does not remain significant after Holm adjustment. The inverse age contrast is nominally significant for moderate-or-worse disability, while its joint Holm-adjusted test is not. The two specifications change both the adjustment set and the estimator, so shifts cannot be attributed solely to additional covariate adjustment. Neither specification establishes causal effects or eliminates residual confounding.

The main five-variable model describes demographic, employment, anthropometric, and behavioral characteristics with a limited number of parameters. No prospectively documented covariate-selection protocol was available, and this exploratory specification does not provide comprehensive confounding control. The expanded sensitivity analysis assesses adjustment for all 11 measured factors without significance-based screening.

Method reference [27]: Kosmidis, I.; Firth, D. Multinomial logit bias reduction via the Poisson log-linear model. Biometrika 2011, 98, 755–759. https://doi.org/10.1093/biomet/asr026.

Part D. Sensitivity model using original workplace and BMI categories

Reference outcome: no disability. HC0 robust standard errors. The detailed results demonstrate heterogeneity masked by the pooled workplace and BMI variables; estimates for small subgroups are imprecise and should not be interpreted as stable subgroup effects.

Outcome contrast	Predictor	Adjusted RRR (95% CI)	*p*
Mild vs no disability	Female vs male	2.42 (1.49–3.94)	<0.001
Mild vs no disability	Age, per year	0.99 (0.92–1.07)	0.785
Mild vs no disability	Ministry of Health hospital vs private rehabilitation	1.29 (0.73–2.29)	0.379
Mild vs no disability	Primary health center vs private rehabilitation	1.35 (0.29–6.35)	0.701
Mild vs no disability	University hospital vs private rehabilitation	1.13 (0.31–4.15)	0.857
Mild vs no disability	Military hospital vs private rehabilitation	0.55 (0.21–1.41)	0.212
Mild vs no disability	Underweight vs normal BMI	0.95 (0.38–2.43)	0.922
Mild vs no disability	Overweight vs normal BMI	2.52 (1.50–4.24)	<0.001
Mild vs no disability	Obese vs normal BMI	0.35 (0.08–1.62)	0.181
Mild vs no disability	Smoking: yes vs no	1.56 (0.79–3.10)	0.204
Moderate-or-worse vs no disability	Female vs male	1.79 (0.75–4.26)	0.188
Moderate-or-worse vs no disability	Age, per year	0.86 (0.74–1.00)	0.044
Moderate-or-worse vs no disability	Ministry of Health hospital vs private rehabilitation	3.38 (1.31–8.71)	0.012

**Outcome contrast**	**Predictor**	**Adjusted RRR (95% CI)**	***p***
Moderate-or-worse vs no disability	Primary health center vs private rehabilitation	12.98 (2.51–67.11)	0.002
Moderate-or-worse vs no disability	University hospital vs private rehabilitation	8.69 (2.13–35.48)	0.003
Moderate-or-worse vs no disability	Military hospital vs private rehabilitation	2.31 (0.69–7.75)	0.175
Moderate-or-worse vs no disability	Underweight vs normal BMI	1.01 (0.22–4.58)	0.986
Moderate-or-worse vs no disability	Overweight vs normal BMI	2.49 (1.07–5.77)	0.033
Moderate-or-worse vs no disability	Obese vs normal BMI	1.51 (0.18–12.38)	0.704
Moderate-or-worse vs no disability	Smoking: yes vs no	2.54 (0.85–7.63)	0.097
