# Supplementary material for: Neck Disability, Associated Factors, and Quality of Life Among Physiotherapists in Saudi Arabia
Source: J Clin Med. 2026 Sep 23;15(19):7390. doi: 10.3390/jcm15197390 (PMC13665763; doi:10.3390/jcm15197390)
Supplement: Supplementary file 1 [file jcm-15-07390-s001.zip › Table_S3_QoL_Pairwise_Comparisons.pdf]

Supplementary Table S3. Pairwise WHOQOL-BREF comparisons between NDI levels

Positive rank-biserial r indicates higher scores in Group 1. Holm adjustment was applied within each domain across its three pairwise comparisons.

Domain	Group 1	Group 2	Mann–Whitney U	Raw *p*	Holm *p*	Rank-biserial r
Physical health	No disability	Mild disability	19154.0	<0.001	<0.001	0.444
Physical health	No disability	Moderate-or-worse disability	5859.5	<0.001	<0.001	0.681
Physical health	Mild disability	Moderate-or-worse disability	4189.5	0.002	0.002	0.310
Psychological health	No disability	Mild disability	17744.5	<0.001	<0.001	0.338
Psychological health	No disability	Moderate-or-worse disability	5276.0	<0.001	<0.001	0.514
Psychological health	Mild disability	Moderate-or-worse disability	3680.5	0.136	0.136	0.151
Social relationships	No disability	Mild disability	16250.0	<0.001	0.001	0.225
Social relationships	No disability	Moderate-or-worse disability	4481.5	0.004	0.008	0.286
Social relationships	Mild disability	Moderate-or-worse disability	3340.0	0.659	0.659	0.044
Environment	No disability	Mild disability	16959.0	<0.001	<0.001	0.279
Environment	No disability	Moderate-or-worse disability	5322.5	<0.001	<0.001	0.527
Environment	Mild disability	Moderate-or-worse disability	3841.5	0.047	0.047	0.201
