# Supplementary material for: Neck Disability, Associated Factors, and Quality of Life Among Physiotherapists in Saudi Arabia
Source: J Clin Med. 2026 Sep 23;15(19):7390. doi: 10.3390/jcm15197390 (PMC13665763; doi:10.3390/jcm15197390)
Supplement: Supplementary file 1 [file jcm-15-07390-s001.zip › Table_S4_Age_Stability_Analyses.pdf]

Supplementary Table S4. Age-stability analyses

Age analyses are exploratory. The exact 2,000 bootstrap resampling indices and fitted age coefficients are supplied as Files S3 and S2, respectively. The centered quadratic term was not statistically significant in either severity contrast.

Analysis	Outcome contrast	Age term	RRR / interval	*p*
Primary continuous-age model	Mild vs no disability	Age per year	0.99 (0.92–1.07)	0.866
Primary continuous-age model	Moderate-or-worse vs no disability	Age per year	0.84 (0.74–0.95)	0.008
Categorical-age sensitivity	Mild vs no disability	Age 27–29 vs 21–26	0.77 (0.45–1.31)	0.337
Categorical-age sensitivity	Mild vs no disability	Age 30–42 vs 21–26	1.06 (0.60–1.88)	0.847
Categorical-age sensitivity	Moderate-or-worse vs no disability	Age 27–29 vs 21–26	0.61 (0.28–1.34)	0.220
Categorical-age sensitivity	Moderate-or-worse vs no disability	Age 30–42 vs 21–26	0.44 (0.16–1.22)	0.114
Quadratic-age sensitivity	Mild vs no disability	Centered age	0.97 (0.89–1.05)	0.412
Quadratic-age sensitivity	Mild vs no disability	I(Centered age^2)	1.01 (0.99–1.03)	0.265
Quadratic-age sensitivity	Moderate-or-worse vs no disability	Centered age	0.83 (0.73–0.94)	0.002
Quadratic-age sensitivity	Moderate-or-worse vs no disability	I(Centered age^2)	1.01 (0.99–1.03)	0.311
2,000-resample bootstrap	Mild vs no disability	Age per year	Percentile RRR interval 0.92–1.08	—
2,000-resample bootstrap	Moderate-or-worse vs no disability	Age per year	Percentile RRR interval 0.71–0.94	—
